# Two Main Cellular Components in Rheumatoid Arthritis: Communication Between T Cells and Fibroblast-Like Synoviocytes in the Joint Synovium

**DOI:** 10.3389/fimmu.2022.922111

**Published:** 2022-07-01

**Authors:** Jiajie Tu, Wei Huang, Weiwei Zhang, Jiawei Mei, Chen Zhu

**Affiliations:** ^1^Institute of Clinical Pharmacology, Anhui Medical University, Key Laboratory of Anti-Inflammatory and Immune Medicine, Ministry of Education, Anhui Collaborative Innovation Center of Anti-Inflammatory and Immune Medicine, Hefei, China; ^2^Department of Gynecology, The First Affiliated Hospital of Shenzhen University, Health Science Center, Shenzhen Second People’s Hospital, Shenzhen, China; ^3^Department of Orthopaedics, The First Affiliated Hospital of USTC, Division of Life Sciences and Medicine, University of Science and Technology of China, Hefei, China; ^4^Departments of Geriatrics, The First Affiliated Hospital of USTC, Division of Life Sciences and Medicine, University of Science and Technology of China, Hefei, China

**Keywords:** RA, T cells, FLS, cellular interaction, therapy

## Abstract

Rheumatoid arthritis (RA) is a chronic autoimmune disease that endangers the health of approximately 1% of the global population. Current RA medications on the market mainly include non-steroidal anti-inflammatory drugs, biological agents, and disease-modifying drugs. These drugs aim to inhibit the overactivated immune response or inflammation of RA, but they cannot cure RA. A better understanding of the pathogenesis of RA will provide a new understanding to search for RA targets and for drug development. The infiltration of T cells and hyper-proliferation of fibroblast-like synoviocytes (FLS) in the synovium of patients with RA are significantly upregulated. Furthermore, the abnormal activation of these two types of cells has been confirmed to promote development of the course of A by many studies. This article systematically summarizes the interactions between T cells and FLS in RA synovial tissues, including one-way/mutual regulation and direct/indirect regulation between the two. It further aims to investigate the pathogenesis of RA from the perspective of mutual regulation between T cells and FLS and to provide new insights into RA research.

## 1 Introduction

In the past few decades, extensive research has been conducted to illustrate the important role of T lymphocytes (T cell) in rheumatoid arthritis (RA) (1). In RA, T cell can interact with antigen-presenting cells, including dendritic cell, macrophage, B lymphocyte (B cell), and even non-professional antigen-presenting cell, such as fibroblast-like synoviocyte (FLS). During T cell activation, CD4^+^ T cells initially form contacts with human leukocyte antigen (HLA) or major histocompatibility class II (MHC-II) molecules and co-stimulatory molecules (e.g., CD28) of other cells, leading to the maturation of CD4^+^ cells (2). Subsequently, antigens presented by other cells promote the activation of CD8^+^ T cells, further exacerbating inflammation in RA (3). The interaction between T cells and other cellular components is a key factor in RA pathogenesis.

Apart from immune cells, non-immune cells of target organs also play a vital role in various autoimmune diseases, forming the foundation of the pathogeneses of these diseases (4). FLS are a special type of non-immune cells present in synovial tissue around joints. FLS play an important destructive role in the pathogenesis of RA; specifically, the numbers of FLS significantly increase and become an important part of the destructive pannus that characterizes the synovial membrane of patients with RA. In addition, FLS in RA exhibit an aggressive phenotype and mediate inflammation and joint destruction. Therefore, cellular crosstalk between FLS and other cellular components might also play an important role in RA, especially in the pathology of the joint synovium.

In this review, we summarize the pathophysiological features of T cells and FLS, which are two important cellular types in the joint synovium of patients with RA, at the functional and molecular level. Further, we outline the interactions between T cells and FLS in RA. Finally, we summarize the potential therapeutic options by explaining the roles of these cells in RA.

## 2 Indirect Regulation of T Cells in RA by FLS

### 2.1 Indirect Promotion of T Cell Survival and Chemotaxis by FLS in RA

**Figure 1:**
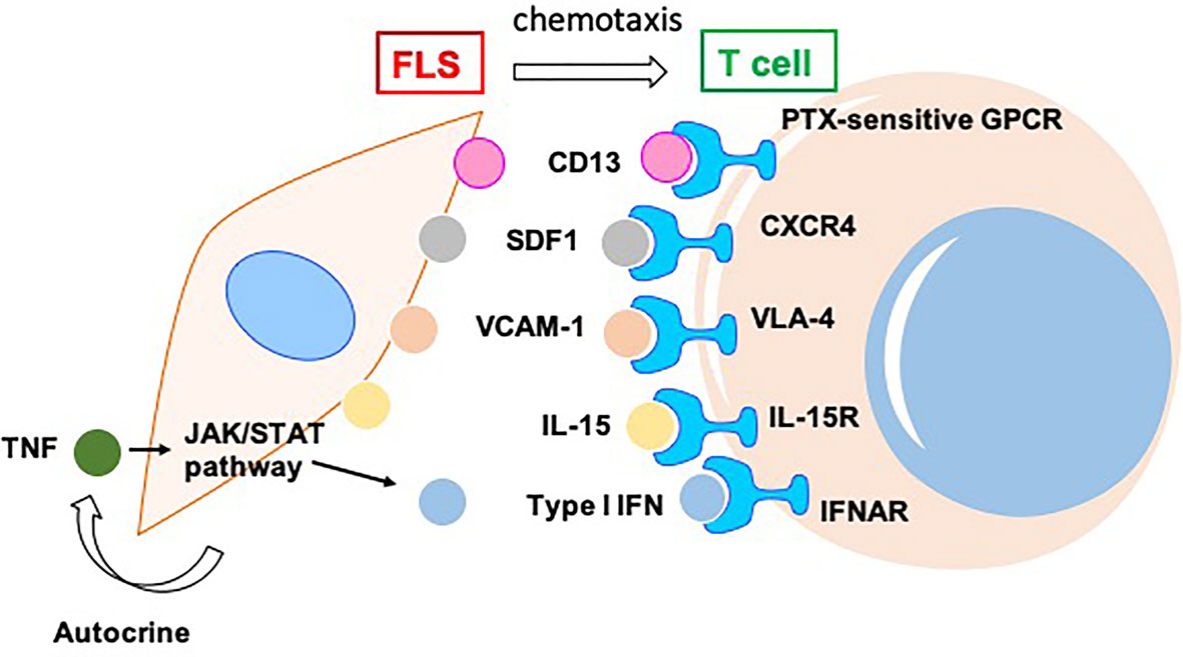
RA-FLS indirectly promotes survival and chemotaxis of T cells in joint synovium of patients with RA *via* by producing various chemokines, including CD13, SDF1, VCAM1, IL15, TNF, and type I IFN. SDF, Stromal cell-derived factor; VCAM, Vascular cell adhesion molecule; VLA,Integrins alpha; CXCR, CXC motif chemokine receptor; IL, interleukin; JAK, Janus kinase; STAT, signal transducer and activator of transcription; TNF, tumor necrosis factor; IFN, interferon.

RA is an autoimmune disease associated with severe synovitis and the destruction of bone and cartilage. In the synovial tissues of patients with RA, T cells can interact with other immune cells, such as macrophages and B cells, and other non-immune cells, including FLS, leading to T cell recruitment, activation, and cytokine production (5). This section focuses on these functions of T cells mediated by FLS-secreted chemokines (**Figure 1**).

CD13 released from FLS induces chemotaxis and T cell activation through a pertussis toxin-sensitive G protein-coupled receptor in RA (6). FLS-derived stromal cell-derived factor (SDF)-1 and vascular cell adhesion molecule (VCAM)-1 recruit T cells *via* their corresponding receptors, CXC motif chemokine receptor (CXCR)-4 and integrins alpha (VLA)-4, respectively, in RA (7). FLS can produce an abundance of proinflammatory cytokines in RA joints. For example, interleukin (IL)-15 is mainly responsible for local T cell activation and proliferation (8). The action of FLS-derived IL-7 is essential for lymphoid neogenesis in the RA synovium (9). The Janus kinase (JAK)/signal transducer and activator of transcription (STAT) pathway in FLS is indirectly activated by the tumor necrosis factor (TNF) through the autocrine expression of type I interferon (IFN), resulting in IFN-α/β receptor engagement and the production of chemokines by T cells, which play a role in the effects of the JAK inhibitor CP-690550 (tofacitinib) in the treatment of RA. The reduction of chemokine synthesis mediated by FLS limits the recruitment of T cells and other infiltrating leucocytes (10).

### 2.2 Indirect Regulation of CD4^+^ T Cell Differentiation in RA by FLS

**Figure 2:**
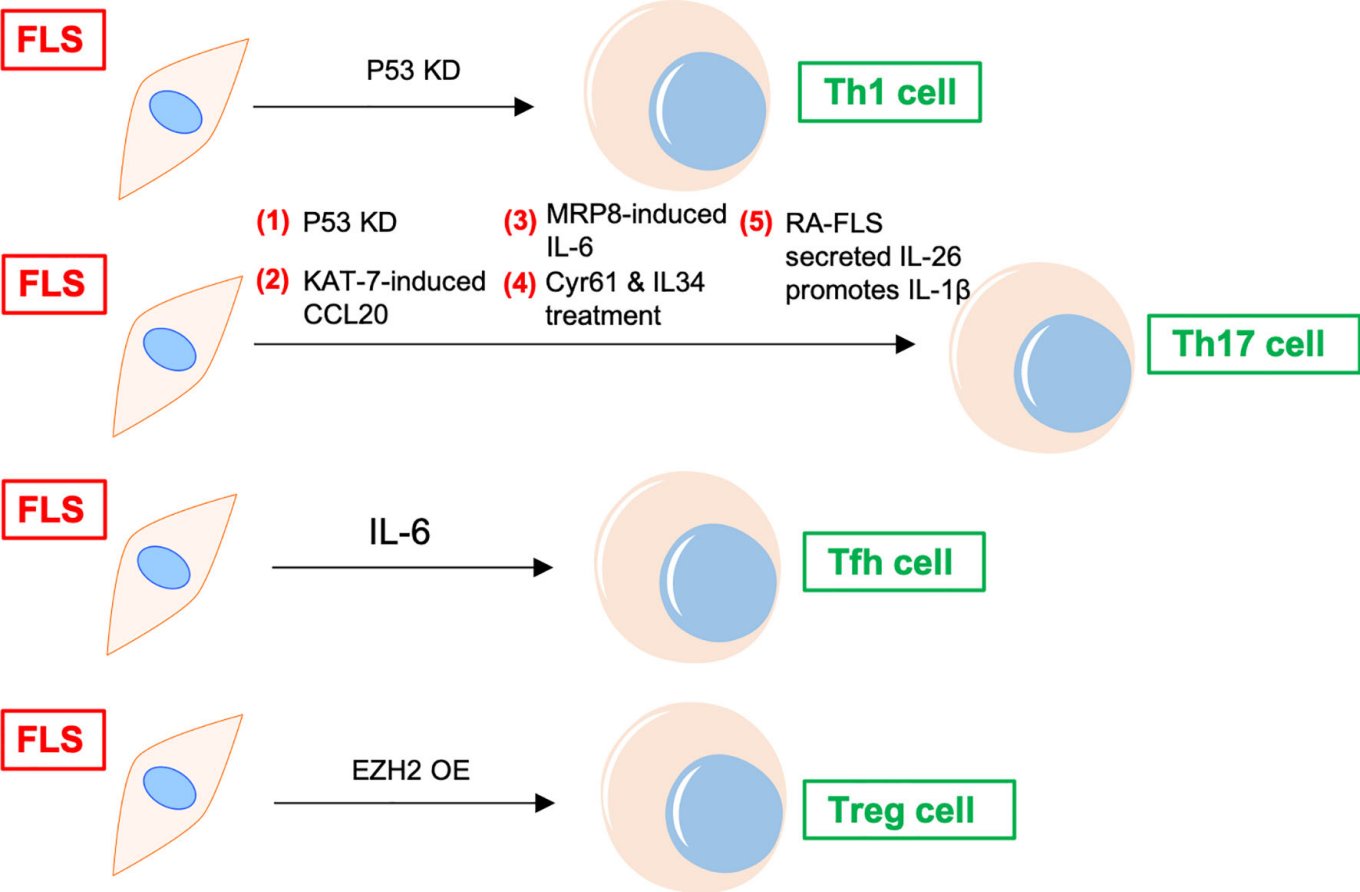
FLS regulates differentiation of CD4^+^ T cells in RA. P53 KD and EZH2 OE promote Th1 and Treg differentiation of T cells in RA synovium, respectively. FLS promotes Tfh differentiation of T cells *via* IL-6. FLS directly induces Th17 differentiation through cytokines, including CCL20, MRP8, IL-6, and IL-26, etc. KD, knockdown; OE, overexpression; Tfh, T follicular helper; CCL, C–C motif chemokine ligand; MRP, myeloid-related protein; TLR, Toll-like receptor; Cyr61, cysteine-rich protein 61; EZH, enhancer of zeste homolog; Treg, regulatory T cells.

In addition to recruiting and activating T cells, FLS can also promote the differentiation of proinflammatory subtypes and inhibit the differentiation of anti-inflammatory subtypes of T cells in the synovial joints of patients with RA (11) (**Figure 2**). FLS co-cultured with peripheral blood mononuclear cells (PBMCs) increase peripheral T follicular helper (Tfh) cell (CD4^+^CXCR5^+^ICOS^+^) count in patients with RA (12). Adiponectin-stimulated FLS can also promote Tfh generation, predominantly *via* IL-6 production in RA (13). P53 abrogates FLS-induced Th1 and Th17 cell differentiation in RA (14). Upregulated KAT7, an H4-specific histone acetylase in FLS, promotes Th17 cell differentiation in RA by inducing C–C motif chemokine ligand (CCL) 20 expression and the p44/42 mitogen-activated protein kinase pathway (15). Further, myeloid-related protein (MRP)8/MRP14 is an endogenous Toll-like receptor 4 (TLR4) ligand. MRP8 produced by FLS can promote Th17 differentiation by enhancing the expression of IL-6 in RA. MRP8 induces IL-6 secretion in FLS *via* TLR4/phosphoinositide 3-kinase (PI3K)/nuclear factor kappa B (NF-κB) and mitogen-activated protein kinase signaling pathways in RA (16). Moreover, IL-34/colony stimulating factor 1 receptor (CSF-1R) axis-induced FLS upregulate Th17 production through increased IL-6 in RA (17). In addition, cysteine-rich protein 61 (Cyr61) induces IL-6 production by FLS, promoting Th17 differentiation *via* the Avb5/Akt/NF-kB signaling pathway in RA (18). Co-cultured FLS enhance PBMC-secreted IL-17-A, IL-6, IFNγ, and IL-1β production in RA (19). FLS and macrophages are the main sources of IL-26 in RA joints. IL-26 induces production of the proinflammatory cytokines IL-1β, IL-6, and TNF-α in monocytes. IL-26-stimulated monocytes selectively promote the generation of RORγt^+^ Th17 cells through IL-1β secretion by monocytes. Therefore, IL-26 is constitutively produced by FLS, induces proinflammatory cytokines in myeloid cells, and promotes Th17 cell differentiation in RA (20). Synovial fluid and FLS from patients with RA suppress enhancer of zeste homolog 2 (EZH2) expression in CD4^+^ T cells. EZH2 deficiency attenuates regulatory T cells (Treg) differentiation in RA (21). Overall, IL-6 seems to be a key inflammatory factor released by FLS in RA. Thus, FLS indirectly affect the differentiation of T cells in the synovial joints of patients with RA through IL-6, promoting the differentiation of Th17 and Tfh cells.

## 3 Indirect Regulation of FLS in RA by T Cells

### 3.1 Promotion of FLS Inflammatory Phenotypes in RA *via* Cytokines From T Cells

#### 3.1.1 Indirect Effects of Th17 Cells on FLS in RA

**Figure 3:**
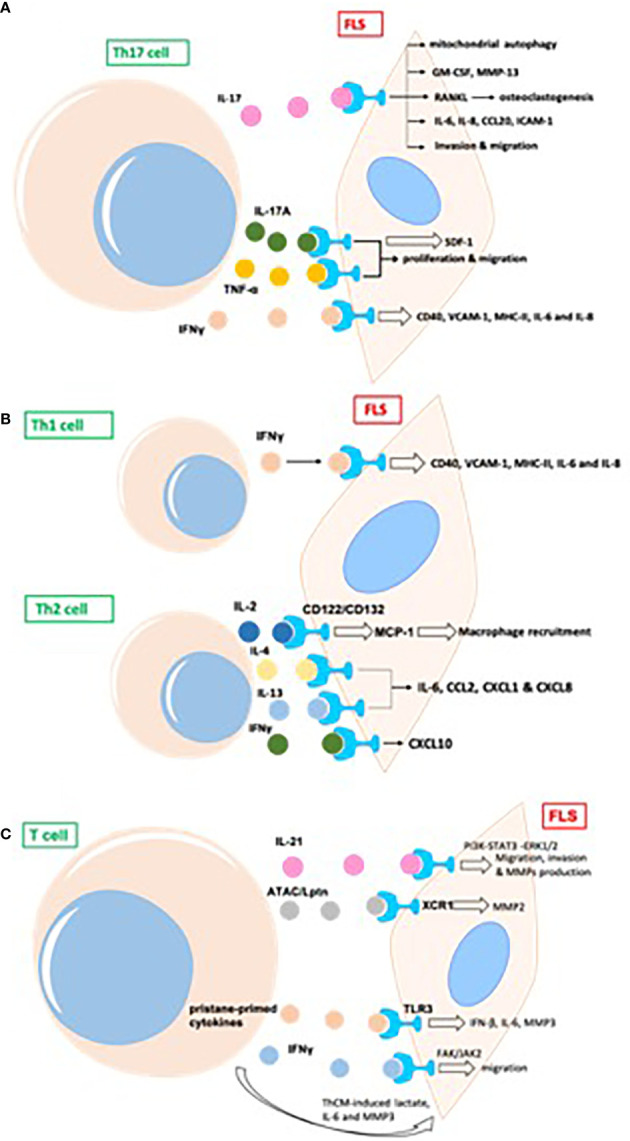
T cells promote inflammatory phenotype of FLS in RA, mainly including proliferation, migration, invasion, and production of proinflammatory cytokines and destructive MMPs in joint synovium. **(A)** The indirect effects of Th17 cells on FLS in RA; **(B)** The indirect effects of Th1/Th2 cells on FLS in RA; **(C)** The indirect effects of other T cells on FLS in RA. GM-CSF, granulocyte-macrophage colony-stimulating factor; MMP, matrix metalloproteinase; RANKL, receptor activator of NF-κB ligand; TRAF, TNF receptor-associated factor; AP, activator protein; ICAM, intercellular adhesion molecule; MIF, migration inhibitory factor; ATAC, activation-induced, T cell-derived, and chemokine-related cytokine; Lptn, lymphotactin; FAK,focal adhesion kinase.

Different subtypes of CD4^+^ T cells can be detected in the synovial joints of patients with RA (22). Th17 promotes the development of RA and is an important aspect of the proinflammatory function of FLS. Transcripts of *IL-17R*, as well as those of *IL-17RB*, *C*, and *D*, have been previously detected in the FLS of patients with RA (23) (**Figure 3A**).

Th17 cells induce secretion of the cytokine granulocyte-macrophage colony-stimulating factor (GM-CSF) in synovial stroma and innate lymphoid cells to initiate and augment autoimmune arthritis (24). Th17 cells and IL-17 increase autophagy of FLS by causing mitochondrial dysfunction in RA (25). The blockade of IL-17 alleviates inflammation in rat arthritis and matrix metalloproteinase (MMP)-13 expression from FLS (26). In addition, IL-17-induced receptor activator of NF-κB ligand (RANKL) expression is decreased by the inhibition of Act1, TNF receptor-associated factor 6 (TRAF6), and activator protein (AP)-1. In the absence of RANKL, IL-17-prestimulated FLS induce osteoclastogenesis from monocytes, which is repressed by the inhibition of TNF-α (27). FLS express two types of phospholipase D, namely PLD1 and PLD2. PLD regulates the Th17-promoted production of proinflammatory cytokines by FLS (28). The dihydroartemisinin derivative DC32 inhibits the Th17-induced invasion and migration of FLS by decreasing the secretion of MMPs (MMP-2, MMP-3) *in vitro* (29).

Th17-cell-secreted IL-17A and TNF-α have synergistic effects on promoting the production of inflammatory cytokines in FLS from patients with RA, the human leukemia cell line THP-1, and the rheumatoid synovial fibroblast cell line MH7A. IL-17A and TNF-α also promote the proliferation and migration of MH7A cells. However, a novel dual targeting fusion protein (targeting TNF-α and IL-17A) was found to be more efficient in inhibiting these synergistic effects when compared to the effects of etanercept (30). Stromal cell-derived factor 1 (SDF-1) is overproduced in RA FLS, and IL-17 upregulates the expression of SDF-1 in RA FLS *via* pathways mediated by PI3K, NF-κB, and AP-1 (31).

#### 3.1.2 Indirect Effects of Th1/Th2 Cells on FLS in RA

Apart from Th17, Th1 and CXCR3^+^ Th2 phenotypes are the main subtypes of T helper cells in the synovium of patients with RA; IL-4 and IL-13 induce FLS to produce a series of inflammatory cytokines, such as IL-6, CCL2, CXCL1, and CXCL8, whereas IFNγ promotes the expression of CXCL10 (32). Both Th1 and Th17 cells produce IL-17 and IFNγ. The expression of CD40, intercellular adhesion molecule 1 (ICAM-1), and MHC-II in FLS is upregulated upon co-culture with Th1 cells, whereas Th17 cells induce only ICAM-1 in FLS. Both T cell subsets promote the production of IL-6 and IL-8 by FLS from patients with RA (33). T cell-derived IL-2 might activate FLS (via IL-2 receptor (CD122) and (CD132) chains) to produce MCP-1, thus recruiting macrophages into the rheumatoid synovium and promoting inflammation (34). Both Th1 and Th2 cells express macrophage migration inhibitory factor (MIF). MMPs are induced by FLS after co-culture with Th1 and Th2 cells, and activated T helper cells are more effective than resting cells. The neutralization of MIF by an anti-MIF antibody leads to the downregulation of MMP in both Th1- and Th2-stimulated FLS (35) (**Figure 3B**).

#### 3.1.3 Indirect Effects of Other T Cells on FLS in RA

IL-21 is produced primarily by CD4^+^ T cells and natural killer T cells. IL-21 induces the migration, invasion, and production of MMPs (MMP-2, MMP-3, MMP-9, MMP-13) in FLS from patients with RA (36). IL-21 promotes activation of the PI3K, STAT3, and ERK1/2 pathways in FLS, and the inhibition of these pathways attenuates IL-21-mediated migration and the production of MMPs (36). The percentage of T cells from the synovial fluid in patients with RA is upregulated relative to that in patients with psoriatic arthritis (37). The proportion of IL-21^+^CD4^+^ T cells from peripheral blood in patients with RA is positively associated with IgM-rheumatoid factor, serum anticyclic citrullinated peptide antibodies, and disease activity score 28 (DAS28). IL-21 expression in synovial fluid is correlated with MMPs; IL-21 significantly induces the production of MMPs in synovial biopsies from patients with RA (37). CD4^+^IL-21^+^ T cells sorted from synovial fluid promote the secretion of MMPs by FLS to a greater extent than medium or CD4^+^IL-21^−^ T cells in an *in vitro* co-culture system. The blockage of IL-21 and TNF leads to the downregulation of MMPs from FLS (37).

In phorbol myristate acetate/ionomycin-stimulated PBMCs, activation-induced, T cell-derived, and chemokine-related cytokine (ATAC)/lymphotactin (Lptn) is detected in CD8^+^ T cells and is upregulated in CD4^+^CD28^−^ T cells from patients with RA as compared with their levels in healthy controls (38). FLS express the ATAC/Lptn receptor XCR1 in the RA synovium. ATAC/Lptn leads to the marked downregulation of MMP2 production in FLS (38). TLR3 is induced in the synovium of rats with pristane-induced arthritis (39). In addition, activation of the TLR3 signaling pathway promotes the development of this arthritis model. Interestingly, pristane-primed T cell-derived cytokines further promote FLS activation (39).

IFNγ produced by T cell stimulation promotes the phosphorylation of focal adhesion kinase (FAK)-Y925, which is important for cell migration (40). SiRNA-mediated knockdown of JAK2, but not JAK1, substantially suppresses FAK activation *via* IFNγ. IFNγ-induced FAK activation and invasion of FLS are also blocked by baricitinib (JAK inhibitor) (40). Soluble mediators released by Th cells drive synovial fluid towards a glycolytic and proinflammatory phenotype. Targeting JAKs or glycolytic enzymes modulates synovial fluid glucose metabolism and decreases the secretion of IL-6 and MMP3 (41). Therefore, targeting glycolytic pathways represents a potential therapeutic strategy to treat inflammation in synovial fluid (41) (**Figure 3C**).

## 4 Direct Interaction Between FLS and T Cells in RA

### 4.1 Direct Regulation of T cells by FLS in RA

**Figure 4:**
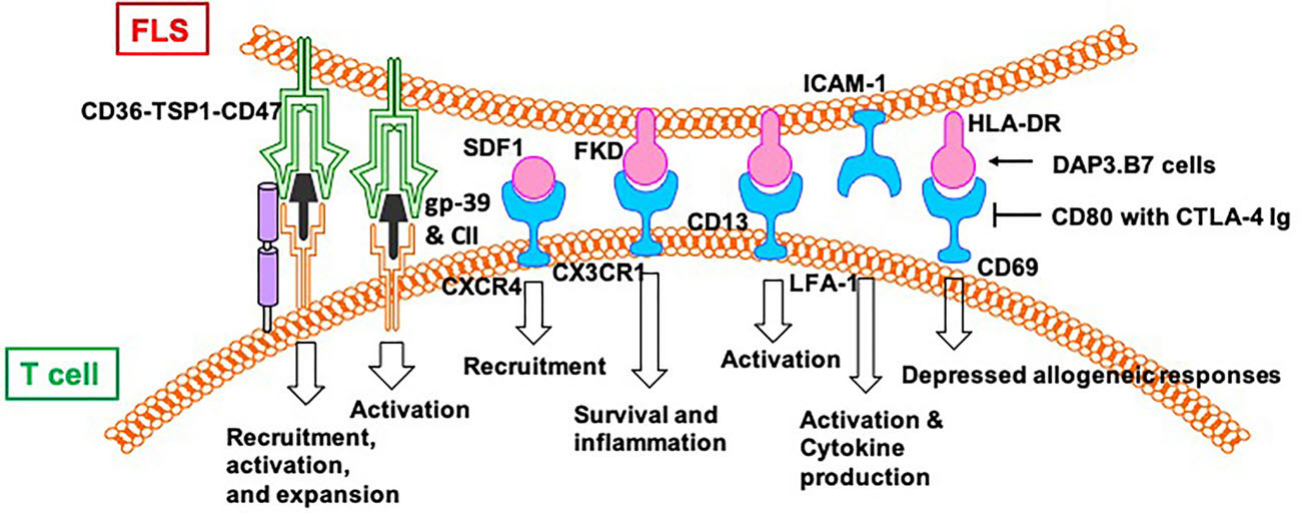
FLS directly regulate functions of T cells in RA.

In addition to indirect regulation through cytokines and chemokines, there is a direct interaction between T cells and FLS in the synovium of patients with RA. Different antigen-presenting cells, including B cells, macrophages, and dendritic cells, interact directly with T cells. FLS, as non-immune cells, also have antigen-presenting capabilities. This section summarizes the direct communication between T cells and FLS in the synovium of patients with RA (**Figure 4**).

Despite not being professional antigen-presenting cells, FLS can also present peptides, such as human cartilage gp-39 and human type II collagen (CII), derived from autoantigens discovered in the joint tissues of patients with RA, to activated T cells *in vitro* in an IFN-dependent and MHC-restricted manner (42). Cell–cell contact between T cells and FLS induce the lymphocytic expression of aminopeptidase N/CD13 and results in lymphocytic activation (43). Both FLS (production of SDF-1) and CD8/CD4^+^ T cells (expression of CXCR4) play a positive role in the recruitment of T cells in the joint synovium (44). CD4^+^ T cells abnormally express CX3CR1 in the synovium of patients with RA. Fractalkine (FKN) induces the adhesion of CD4^+^ T cells and survival signals and co-stimulates the secretion of inflammatory cytokines and granules. CD4^+^ T cells accept primary stimulatory and co-stimulatory signals from non-professional antigen-presenting cells, such as FLS, in the RA synovial microenvironment (45).

A previous study showed the effects of FLS on the recruitment, activation, and expansion of T cells in RA in a CD47-TSP1 (thrombospondin-1)-dependent manner (46). TSP1-mediated co-stimulation is achieved through its independent interaction with CD36 on antigen-presenting cells and with CD47 on T cells. A CD47–TSP1–CD36 trimolecular complex is a new co-stimulatory pathway that represses the activation of T cells. Because the lesions in rheumatoid synovitis are sites of antigenic recognition, the identification of TSP1 on antigen-presenting cells such as FLS suggests the central role of TSP1 in the expansion of T cells in RA (47). Direct contact between T cells and FLS induces the expression of HLA-DR on FLS and CD69 on T cells in an allogeneic and autologous manner. The addition of DAP3.B7 cells to co-cultures of T cells and FLS alleviates the repressed allogeneic activation of T cells (48). The allogeneic response by T cells to FLS in the presence of DAP3.B7 cells can be blocked by inhibiting CD80 with CTLA-4 Ig (48). Strong expression of B7-H3 was detected on FLS in synovial tissue of a patient with RA (49). Cells expressing B7-H3 are distinct from but very close to cells expressing CD45, CD3, and CD20. In addition, FLS and T cell co-cultures show localization of B7-H3 in the contact section between them but this is distinct from CD11a/CD18 (LFA-1)^+^ T cells and ICAM-1^+^ FLS. Blocking B7-H3 on FLS affects the interactions between FLS and T cells. Resting T cells have upregulated IL-2, TNF-α, and IFN-γ, whereas cytokine-activated T cells exhibit downregulated cytokine production. However, cytokine production by T cells activated *via* TCR is not affected by B7-H3 (49).

### 4.2 Direct Regulation of FLS by T Cells in RA

Direct contact between activated CD4^+^ T cells and an FLS-facilitated hGITR–GITRL interaction lead to the upregulation of MMP-13 (50). CII-reactive T cells induce the secretion of chemokines (IL-8, MCP-1, and MIP-1α) through interactions with FLS in RA joints, which is mediated by CD40L–CD40 communication (51). FKN–CX3CR1 receptor–ligand interactions affect FLS growth and T cell functions. FLS promote autocrine growth by releasing FKN and triggering the activity of CX3CR1. This growth-promotion loop is amplified by CX3CR1^+^ T cell-produced TNF-α upon stimulation by FKN^+^ FLS (52).

Mutual activation of T cells and FLS results in increased proliferation and expression of ICAM-1 and VCAM-1 by both CD4^+^ T cells and FLS (53). The interaction between CD4^+^ T cells and FLS results in the upregulation of TNF-α, IFN-γ, and IL-17A from CD4^+^ T cells and the secretion of other cytokines, including IL-6, IL-8, and vascular endothelial growth factor (VEGF). Moreover, CD4^+^ T cells cultured in conditional medium promote invasiveness and glycolysis in FLS while repressing oxidative phosphorylation, with the effects paralleled by induced glucose transporters GLUT1 and GLUT3, key glycolytic enzymes GSK3A, HK2, LDHA, and PFKFB3, VEGF, and MMPs, which is alleviated by the glycolytic inhibitor 2-DG and adenosine monophosphate analogue 5-aminoimidazole-4-carboxamide ribonucleotide (53).

Co-culture with T cells induces the phosphorylation of protein kinase Akt (Ser473) and downstream mediators, including GSK-3α/β, FoxO1/3a, and mouse double minute (MDM)-2, in FLS from patients with RA (54). Co-cultured T cells also promote the proliferation of FLS and the production of IL-6, which is repressed by blocking antibodies to CD11a and ICAM-2. T cell-mediated phospho-Akt upregulation is unique to FLS because no such effect is observed in B cells and dendritic cells. Selective involvement of the LFA-1–ICAM-2 pathway has been confirmed based on increased ezrin phosphorylation at Tyr353 downstream of ICAM-2, which supports cell survival through Akt activation (54).

**Figure 5:**
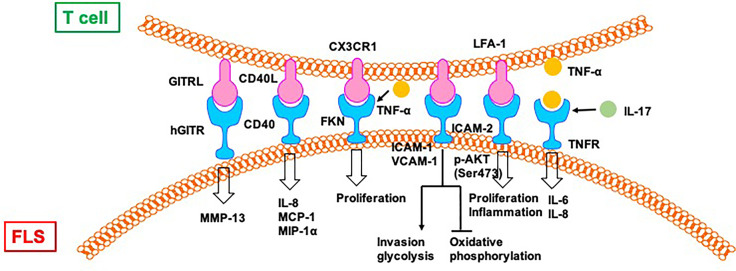
T cells directly regulate FLS in RA. CII:type II collagen; FKN, Fractalkine; TSP, thrombospondin.

The rapid and robust adhesion of cytokine-activated T cells (Tck) and super antigen-activated T cells to FLS leads to flattening and a crawling movement in T cells on the cellular surface of FLS (55). Tck activates FLS to secret IL-6 and IL-8 in a cell contact-dependent manner, which is further activated by IL-17. Antibody blocking of membrane TNF-α on the Tck surface inhibits cytokine production by FLS, demonstrating a novel mechanism of TNF-α during T cells–FLS interactions in the RA synovium (55) (**Figure 5**).

### 4.3 Direct Mutual Regulation of T Cells and FLS in RA

**Figure 6:**
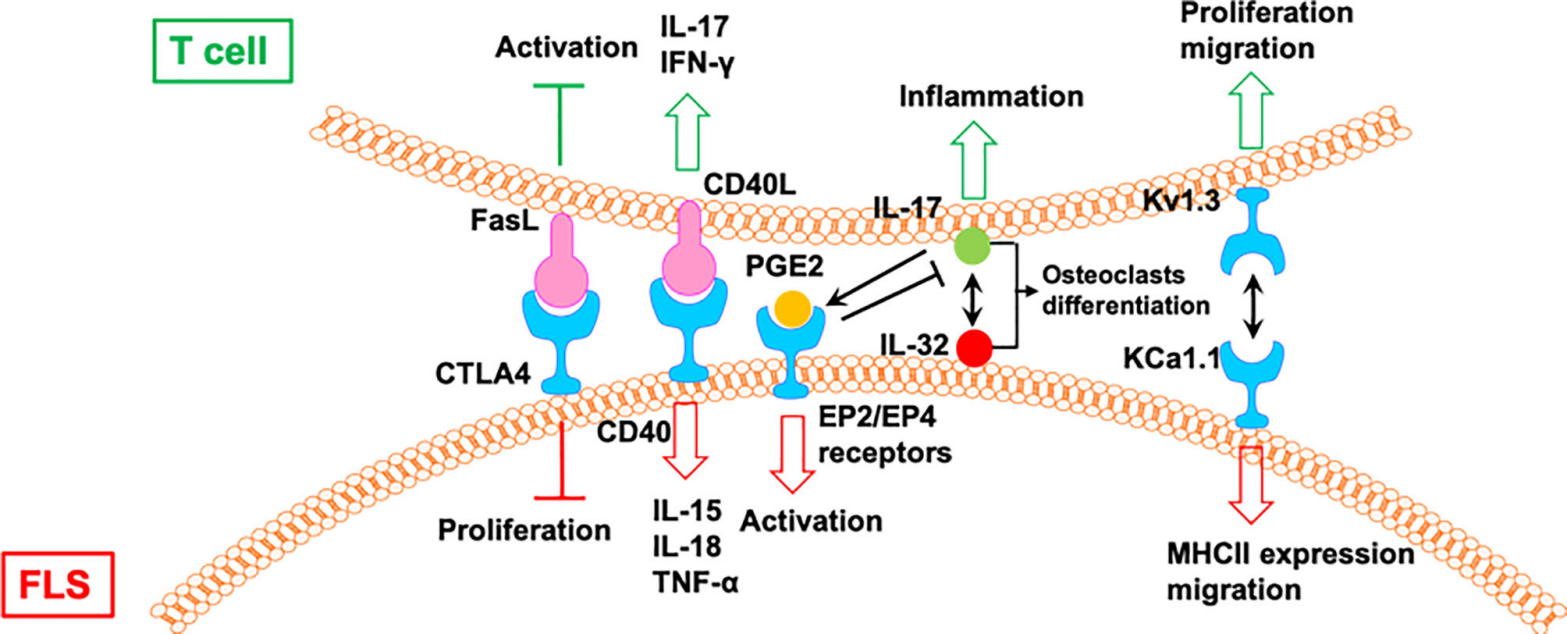
The mutual regulation of T cells and FLS in RA. CIA, collagen-induced arthritis; T_em_: effector memory T; AIA, adjuvant-induced arthritis.

In addition to the one-way direct regulation, there is a direct and mutual crosstalk between T cells and FLS in the synovium of patients with RA. This two-way communication further leads to the development of RA (56) (**Figure 6**). The T cells from patients with RA with a stronger response to CII show higher expression of inflammatory mediators, including IL-15, TNF-α, IFN, and IL-17. When co-incubated with RA FLS, T cells can stimulate the secretion of TNF-α, IL-15, and IL-18 from FLS during CII stimulation. In contrast, T cells also produce higher amounts of IL-17 and IFN-γ during co-culture with RA FLS. The crosstalk between T cells and FLS requires direct cell–cell contact and occurs in a CD40L-CD40 dependent manner (57).

IL-17 induces the expression of IL-32 in FLS from patients with RA, which activates the secretion of IL-17 from CD4^+^ T cells (58). IL-17 and IL-32 are co-localized near tartrate resistant acid phosphatase-positive areas in joints from patients with RA. IL-32 and IL-17 promote osteoclast differentiation in a synergistic manner, and both promote osteoclast resorption *via* RANKL (58).

The interactions between FLS from rats with collagen-induced arthritis (CIA) and rat CCR7^−^ effector memory T (T_em_) cells is regulated by KCa1.1 and Kv1.3 (59). Blocking KCa1.1 on FLS reduces the promoting effects of FLS on the proliferation and migration of T_em_ cells, and blocking Kv1.3 on T_em_ cells reduces the effects of T_em_ cells on the expression of KCa1.1 and MHCII and the invasion of FLS. Furthermore, combination therapies comprising selective KCa1.1 and Kv1.3 inhibitors are more efficacious than monotherapies in alleviating disease features of rat arthritis models (59).

Macrophage-produced PGE2 is a response to IL-17 of T cells, which negatively regulates the expression of TNF-α and IL-17, as well as the TNF-α/IL-1-mediated activation of FLS *via* EP2 and EP4 receptors, resulting in the modulation of proinflammatory cascades in RA (60). A CTLA4-FasL fusion protein suppresses FLS proliferation and the development of adjuvant-induced arthritis (AIA) in rats. However, CTLA4-FasL also acts as an effective inhibitor for T cells; it not only inhibits the activation of T cells but also promotes activated T cell death (61).

## 5 Correlation Between T cells and FLS in RA

The numbers of FLS and T cells in the synovial tissue of patients with RA are closely associated with joint damage (62). RA naiüve T cells share hypermethylation sites with FLS. FLS-representative DNA methylation signatures derived from blood might serve as biomarkers of RA risk or disease status (63). In the following section, we summarize some recent studies reporting that some treatments for RA (**Table 1**) or the regulation of a specific gene/protein (**Table 2**) can affect the function of both T cells and FLS in RA.

**Table 1:** The medicinal treatment regimens that can affect the functions of both T cells and FLS in RA.

Treatment	T cells	FLS	Other	Ref
BBR	BBR inhibits the proliferation of Th17 cells through downregulation of RORγt and promotes the differentiation of Treg cells through induction of Foxp3 activation via up-regulation of AhR and CYP1A1.	BBR inhibited autophagy in AA-FLS mediated through PI3K/Akt signaling via suppression of autophagic elements, p62 sequestration and induction of CHOP. BBR inhibited the proliferation of AA-FLS via promotion of apoptosis.		(64)
Silibinin	Silibinin inhibits Th17 cell differentiation.	Silibinin suppresses cell viability and increases apoptosis of RA-FLS. The production of inflammatory cytokines in RA-FLS and a CIA rat model is inhibited by silibinin.		(65)
Single use or combination treatment with LMT-28 and metformin	Single use or combination treatment with LMT-28 and metformin suppress Th17 differentiation.	Single use or combination treatment with LMT-28 and metformin and IL-6 signaling in FLS.		(66, 67)
LMT-28 and THP combination	LMT-28 and THP combination inhibits Th17 differentiation.	LMT-28 and THP combination suppresses of IL-6 or TNF-induced signaling pathways in RA-FLS.	LMT-28 and THP combination inhibits osteoclastogenesis.	(68)
Diallyl Trisulfide	Diallyl Trisulfide represses Th17 differentiation and has a therapeutic effect of CIA mice.	Diallyl Trisulfide induces FLS apoptosis of CIA mice.		(69)
Oroxylin A	Oroxylin A-treated mice shows an increase in Treg and reduction in Th17 cells in the ILN.	Oroxylin A decreases the secretion of IL-1β and IL-6 from TNFα-stimulated RA FLS *in vitro*.		(70)
Formyl peptide receptor agonist Cpd43	Cpd43 inhibits the expansion, activation and differentiation of arthritogenic effector CD4 T cells.	Cpd43 inhibits proliferation of FLS.		(71)
MTX	In T cell lines, MTX inhibits activation of NF-κB via depletion of BH4 and up-regulation of JNK-dependent p53 activity.	Inhibition of NF-kB activation by MTX is prevented by adenosine receptor antagonists in FLS.		(72)
CP-25	CP-25 decreases the expression of BAFF-R in CD4+ T cells.	CP-25 inhibits the proliferation and cytokine secretion of FLS co-cultured with BAFF-activated CD4+ T cells.		(73)
Bortezomib plus MSC combination	Bortezomib plus MSC combination restores TLR expression and Treg frequency in blood.	Bortezomib plus MSC combination normalizes FLS proliferation, apoptosis and cytokine secretion.	Human UC-MSCs suppress the inflammatory effects of FLSs and T cells of RA.	(74, 75)
monoclonal BsAb (TNF-α and CXCL10)	The BsAb inhibited CXCL10-mediated CD8+ T cell migration.	The BsAb inhibited TNF-α induced ICAM-1 and VCAM-1 in FLS. The BsAb decreased the expression of TNFSF11 and the production of IL-6 in FLS stimulated with TNF-α and CXCL10.		(76)
FL-BsAb1/17	FL-BsAb1/17 could repress the production of IL-1 and IL-17 in T cells.	FL-BsAb1/17 could significantly decrease the production of IL-6 in FLS.		(73)
Huayu Tongbi Fang	Huayu Tongbi Fang decreased GM-CSF production by T cells.	Huayu Tongbi Fang could inhibit FLS activation.		(77)
Clarithromycin	As clarithromycin suppressed HLA-DR and costimulatory molecule expression was enhanced by IFN, autologous T cell proliferation was inhibited by clarithromycin.	Clarithromycin suppressed the production of these cytokines including IL-1, IL-6, IL-8, G-CSF and GM-CSF but did not enhance IL-10 production of FLS.		(78)

Atg5, autophagy-related 5; CHOP, C/EBP homologous protein; BBR, Berberine; BCL, B-cell lymphoma; BAX, Bcl-2 associated X protein; FOXP3, forkhead box P3; AhR, aryl hydrocarbon receptor; CYP1A1, cytochrome P450 family 1, subfamily A, polypeptide 1; SIRT, Sirtuin1; THP, tetrahydropapaverine; MTX, methotrexate; BH4, tetrahydrobiopterin; JNK, Jun-N-terminal kinase; BsAb, bispecific antibody; CP-25, Paeoniflorin-6′-O-benzene sulfonate; BAFF-R,B cell-activating factor, belonging to the TNF family-receptor; UC-MSCs, umbilical cord-derived mesenchymal stem/stromal cells.

**Table 2:** Specific gene/protein that can affect the functions of both T cells and FLS in RA.

Molecule	T cells	FLS	Other	Ref
CCL3	CCL3 could up-regulate CD4+T cells to mediate the inflammatory response.	CCL3 enhanced the expression level of pro-inflammatory cytokines in RA-FLS via activation of the PI3K/AKT signaling pathway.		(79)
IL-21	IL-21 induced RANKL expression in CD4+ T cells from RA patients.	IL-21 induced RANKL expression in RA-FLS.	IL-21 enhanced osteoclastogenesis *in vitro*.	(80, 81)
CTX	CTX suppressed the abnormal increasing of CD4+ T cells/CD8+ T cells ratio, and inhibited T cell proliferation.	CTX inhibited the proliferation of the RA-FLS via suppression of NF-κB signaling pathway.		(79)
rhIL23R-CHR	rhIL23R-CHR decreased secretions of IL-17 and IL-9, whereas FoxP3 was activated in the process in the CIA rats.	rhIL23R-CHR repressed proinflammatory effects on FLS.	synergetic effects with TNF-α	(82)
cDHPS	cDHPS restored the balance of Th17 and Treg cells of CIA mice.	cDHPS reduced the secretion of pro-inflammatory mediators related to FLS activation,	cDHPS repressed angiogenesis, articular cartilage degradation and osteoclast differentiation, inhibited HIF-1α expression and promoted anti-inflammatory mediator release in the joint tissues and serum of CIA mice .	(83)
DP	DP suppressed lipopolysaccharide-induced pro-inflammatory cytokine expression in Jurkat T lymphocytes.	DP inhibited p65 acetylation in MH7A cells, a human RA-FLS line.	DP specifically inhibited the HAT activities of p300/CBP. DP-induced hypoacetylation was accompanied by cytosolic accumulation of p65 and nuclear localization of IKBα. Accordingly, DP treatment inhibited TNFα-stimulated increases in NF-κB function and expression of NF-κB target genes in these cells.	(84)

CTX, Cobrotoxin; cDHPS, Dendrobium huoshanense stem polysaccharide; DP, Delphinidin.

### 5.1 Simultaneous Effect of an RA Drug on T Cells and FLS

IL-21 induces the expression of Beclin-1, autophagy-related 5 (Atg5), and LC3-phosphatidylethanolamine conjugate 3-II (LC3-II) through the inhibition of C/EBP homologous protein (CHOP) in FLS from rats with adjuvant-induced arthritis. Berberine (BBR), an alkaloid derivative predominantly present in Oregon grapes and shoots of barberry, represses FLS autophagy *via* PI3K/Akt signaling by inhibiting autophagic elements, p62 sequestration, and the induction of CHOP. In addition, IL-21 induces the hyper-proliferation of FLS by upregulating the B-cell lymphoma-2 (Bcl-2)/Bcl-2 associated X protein (BAX) ratio, which can be reversed by BBR. IL-21 also promotes CD4^+^ CD196^+^ Th17 cell expansion *via* the PI3K/Akt pathway, and BBR can repress the expansion of Th17 cells by repressing the specific transcriptional factor RORγt in Th17 cells in a PI3K/AKT-dependent manner. Furthermore, BBR promotes the expansion of CD4^+^CD25^+^ Treg cells, which exerts an effect opposite to that of Th17 cells, through induction of a specific Treg transcriptional factor, forkhead box P3 (Foxp3), *via* aryl hydrocarbon receptor (AhR) and the upregulation of cytochrome P450 family 1, subfamily A, polypeptide 1 (CYP1A1) (64).

Silibinin, a natural polyphenolic flavonoid, represses cell proliferation and induces the apoptosis of FLS from patients with RA in an NF-κB pathway-dependent manner. Silibinin also represses Sirtuin1 (SIRT1), and SIRT1 knockdown enhances silibinin-induced apoptosis in FLS. Silibinin also inhibits arthritis development in a CIA rat model and the secretion of inflammatory cytokines in FLS from patients with RA. In addition, it inhibits the differentiation of Th17 cells *in vitro* (65).

Single-use or combination treatment with LMT-28 (a derivative of oxazolidinone) and metformin significantly ameliorates arthritic signs in rats with CIA by suppressing Th17 differentiation and IL-6 signaling in FLS (66, 67). A combination of LMT-28 and tetrahydropapaverine (THP, benzylisoquinoline alkaloid) could attenuate RA through the suppression of Th17 differentiation in T cells and proinflammatory cytokine-induced inflammation in FLS (68). Diallyl trisulfide induces FLS apoptosis, represses Th17 differentiation, and has a therapeutic effect on mice with CIA by blocking NF-κB and Wnt pathways (69). Oroxylin A-treated CIA mice demonstrate an upregulation of Treg cells and downregulation of Th17 cells in the inguinal lymph nodes. Oroxylin A also represses the production of IL-1β and IL-6 from TNFα-stimulated FLS *in vitro* (70). The formyl peptide receptor agonist Cpd43 inhibits the expansion of arthritogenic effector CD4 T cells and FLS and reduces joint damage in CIA and AIA mice (71).

Previous results also showed that methotrexate (MTX) represses the NF-κB pathway in T cells and FLS. In T cell lines, MTX blocks the NF-κB pathway by repressing tetrahydrobiopterin (BH4) and inducing p53 in a Jun-N-terminal kinase (JNK)-dependent manner (72). Levels of phosphorylated RelA are decreased in low-dose MTX-treated patients with RA. However, the MTX-mediated inhibition of the NF-κB pathway is completely prevented by adenosine receptor antagonists in FLS from patients with RA but not *via* BH4 and JNK (72). Clarithromycin represses the secretion of cytokines such as IL-1, IL-6, IL-8, G-CSF, and GM-CSF but does not enhance the production of IL-10 by FLS. As clarithromycin suppresses HLA-DR and co-stimulatory molecule expression is enhanced by IFN, the proliferation of autologous T cells is markedly inhibited by clarithromycin. Clarithromycin exerts a considerable immunosuppressive effect on FLS by inhibiting co-stimulatory molecule expression, cytokine production, and antigen-specific T cell proliferation induced by FLS (78).

The effects of a monoclonal bispecific antibody (BsAb) targeting TNF-α and CXCL10 was also evaluated in RA (76). BsAb repressed the CXCL10-mediated migration of CD8^+^ T cells. Further, the effect of binding of the BsAb to TNF-α was comparable to that of adalimumab; BsAb also repressed TNF-α-mediated cell death and the expression of VCAM-1 and ICAM-1 in FLS. BsAb was also found to inhibit TNFSF11 and IL-6 in TNF-α- and CXCL10-stimulated FLS (76). Another recombinant IgG-like bispecific antibody (FL-BsAb1/17) targeting IL-1β and IL-17A also showed considerable effects for RA treatment, which could repress the secretion of IL-6 in FLS from patients with RA (73). Paeoniflorin-6′-O-benzene sulfonate (CP-25) decreases the expression of B cell-activating factor, belonging to the TNF family-receptor (BAFF-R), in CD4^+^ T cells and represses cell proliferation and cytokine production in FLS co-cultured with BAFF-activated CD4^+^ T cells (85). A Chinese herbal formula, Huayu Tongbi Fang, also represses FL-mediated inflammation in rats by suppressing T cells and FLS-producing GM-CSF (77). Human umbilical cord-derived mesenchymal stem/stromal cells (UC-MSCs) inhibit the inflammatory features of FLS and T cells from patients with RA and alleviate the progression of CIA, implying that UC-MSCs can be used as a potential therapeutic strategy for RA (74). The combination of bortezomib and MSCs rescues TLR expression and the ratio of Treg cells in peripheral blood and normalizes FLS proliferation, apoptosis, and cytokine secretion (75).

### 5.2 Regulation of T cells and FLS by a Common Factor in RA

CCL3 enhances the expression of proinflammatory cytokines (including IL-6, IL-1β, TNF-α, and RANKL) in RA-FLS by activating the PI3K/AKT signaling pathway. Moreover, CCL3 can upregulate CD4^+^ T cells to mediate the inflammatory response in RA (86). Cobrotoxin (CTX) suppresses the abnormal increase in CD4^+^/CD8^+^ T cells and inhibits T cell proliferation. CTX also inhibits the proliferation of cultured FLS by inhibiting the NF-κB signaling pathway (79).

rhIL23R-CHR can be used to inhibit the IL-23-related pathway to explore the role of IL-23 in the dysfunction of Th17/Th9/Treg cells in rats with CIA. CIA rats demonstrate downregulation of the production of IL-9 and IL-17 and upregulation of FoxP3 upon rhIL23R-CHR treatment, implying that IL-23 could alleviate the dysfunctions of Th17/Th9/Treg cells. Furthermore, IL-23 also promotes the proinflammatory features of FLS *in vitro*, showing synergetic outcomes with TNF-α (82).

RANKL is expressed by both FLS and sub-lining T lymphocytes (87). IL-21 promotes RANKL in CD4^+^ T cells from CIA and in CD4^+^ T cells and FLS from patients with RA. IL-21 also induces osteoclastogenesis by inducing RANKL expression in CD4^+^ T cells and FLS *in vitro* (80). Another study detected RANKL^+^ cells in FLS and infiltrating mononuclear cells of synovial tissue of patients with RA (81). Double immunostaining detected RANKL^+^ cells in CD3^+^ and CD4^+^ T cells. RANKL is elevated and osteoprotegerin is lowered in the synovial fluid of patients with RA. The ratio of the concentration of RANKL to that of osteoprotegerin is also upregulated in the synovial fluid of patients with RA compared to that in the synovial fluid of patients administered oroxylin A or with gout. In addition, RANKL^+^ T cells promote osteoclastogenesis from peripheral monocytes. The promoting function of RANKL osteoclastogenesis was confirmed by osteoprotegerin-mediated inhibition in a dose-dependent manner (81).

Dendrobium huoshanense stem polysaccharide (cDHPS) alleviates the imbalance in Th17/Treg cells; represses the production of FLS activation-associated proinflammatory cytokines, damage to articular cartilage, the formation of osteoclasts, and angiogenesis; reduces HIF-1α; and induces anti-inflammatory cytokines in joint synovium and serum of CIA mice (83). Delphinidin represses the histone acetyltransferase activities of p300/CBP and p65 acetylation in MH7A cells, which are a human RA FLS cell line (84). Delphinidin-mediated hypoacetylation is characterized by the cytosolic accumulation of NF-κB activator p65 and nuclear localization of the NF-κB inhibitor IKBα. Delphinidin suppresses the TNF-α-induced upregulation of the NF-κB pathway in MH7A cells. It also represses LPS-induced proinflammatory cytokine production in Jurkat T lymphocytes, implying that a histone acetyltransferase inhibitor can efficiently suppress cytokine-mediated immune responses (84).

## 6 Conclusion and Perspective

T cells and FLS play an important role in the pathogenesis of RA. T cells show a systematic disorder in patients with RA, and FLS promote inflammation and damage the joints locally in the joint synovium of patients with RA. However, since T cells can be recruited to the joint synovium through blood and lymphatic circulation, there is a possibility of interactions between the two cellular components in the joint synovium. Recent publications have confirmed many means of communication between T cells and FLS in the joint synovium in RA, including direct or indirect interactions and one-way or two-way interactions, further amplifying the severity of synovitis. Therefore, blocking this key interaction has the potential to relieve the symptoms of RA or even completely treat RA.

Many agents can directly affect both FLS and T cells in RA. The dual effect of those potential drugs on FLS and T cells presents a promising solution for the treatment of RA and thus, should be further studied in the future. For example, blocking the proinflammatory cytokine (CCL3, IL-21, and IL-23) pathways will block the activation of T cells and FLS-mediated proinflammatory effects because their receptors are commonly expressed on T cells and FLS (88). In addition, for some pathways that can act mutually between T cells and FLS, such as PGE2/EP receptors and Kv1.3/KCa1.1, inhibitors that stop these bidirectional effects should be designed and tested to prevent the cascading proinflammatory effects and relieve the symptoms of RA (89).

But there are still some unsolved issues with the current research, which leads to obstacles to potential application in the future. For example, FLS is not professional APC, and it is not clear whether the molecular mechanism of the signals that activate T cells is exactly the same as that of APCs, and the interaction between FLS and different subtypes of CD4^+^ T cells is also not entirely clear. Secondly, in the joint synovial tissue of RA patients, in addition to FLS and T cells, there are many other important cell types, including B cells, macrophages, etc., and the interaction network between these cells also needs to be further clarified. Finally, the interaction between T cells and FLS in most of the literature mentioned in this review was confirmed by *in vitro* experiments, and whether the same regulatory patterns still exist in the *in vivo* environment require better *in vivo* models to confirm. All of these issues need further in-depth study before clinic application.

## Author Contributions

JT and WH drafted the manuscript. JT, TL, WZ, and CZ revised the manuscript. All authors contributed to the article and approved the submitted version.

## Funding

This work was supported by the National Natural Science Foundation of China (Grant No. 81871788 and 31900616), the Project for Science and Technology leader of Anhui Province (Grant No. 2018H177), the Scientific Research Fund of Anhui Education (Grant No. 2017jyxm1097), the Anhui Provincial Postdoctoral Science Foundation (Grant No. 2019B302), Youth Program of the Provincial Natural Science Foundation of Anhui (2008085MH247), The project of improvement of scientific ability of Anhui Medical University(2020xkjT009) and the Sanming Project of Medicine in Shenzhen (SZSM201812041).

## Conflict of Interest

The authors declare that the research was conducted in the absence of any commercial or financial relationships that could be construed as a potential conflict of interest.

## Publisher’s Note

All claims expressed in this article are solely those of the authors and do not necessarily represent those of their affiliated organizations, or those of the publisher, the editors and the reviewers. Any product that may be evaluated in this article, or claim that may be made by its manufacturer, is not guaranteed or endorsed by the publisher.

## References

[1] Tu J Huang W Zhang W Mei J Zhu C. A Tale of Two Immune Cells in Rheumatoid Arthritis: The Crosstalk Between Macrophages and T Cells in the Synovium. Front Immunol (2021) 12:655477. doi: 10.3389/fimmu.2021.655477 34220809 PMC8248486

[2] Mørch AM Bálint Š Santos AM Davis SJ Dustin ML. Coreceptors and TCR Signaling – the Strong and the Weak of it. Front Cell Dev Biol (2020) 8:597627. doi: 10.3389/fcell.2020.597627 33178706 PMC7596257

[3] Malmström V Trollmo C Klareskog L. Modulating Co-Stimulation: A Rational Strategy in the Treatment of Rheumatoid Arthritis? Arthritis Res Ther (2005) 7 (Suppl 2):S15–20. doi: 10.1186/ar1505 PMC2833979 15833144

[4] Tu J Hong W Zhang P Wang X Körner H Wei W. Ontology and Function of Fibroblast-Like and Macrophage-Like Synoviocytes: How do They Talk to Each Other and Can They Be Targeted for Rheumatoid Arthritis Therapy? Front Immunol (2018) 9:1467. doi: 10.3389/fimmu.2018.01467 29997624 PMC6028561

[5] Schonfeldova B Zec K Udalova IA. Synovial Single-Cell Heterogeneity, Zonation and Interactions: A Patchwork of Effectors in Arthritis. Rheumatol (United Kingdom) (2022) 61:913–25. doi: 10.1093/rheumatology/keab721 PMC8889290 34559213

[6] Morgan R Endres J Behbahani-Nejad N Phillips K Ruth JH Friday SC. Expression and Function of Aminopeptidase N/CD13 Produced by Fibroblast-Like Synoviocytes in Rheumatoid Arthritis: Role of CD13 in Chemotaxis of Cytokine-Activated T Cells Independent of Enzymatic Activity. Arthritis Rheumatol (2015) 67:74–85. doi: 10.1002/art.38878 25219368 PMC4280337

[7] Bryant J Ahern DJ Brennan FM. CXCR4 and Vascular Cell Adhesion Molecule 1 are Key Chemokine/Adhesion Receptors in the Migration of Cytokine-Activated T Cells. Arthritis Rheum (2012) 64:2137–46. doi: 10.1002/art.34394 22275188

[8] Harada S Yamamura M Okamoto H Morita Y Kawashima M Aita T. Production of Interleukin-7 and Interleukin-15 by Fibroblast-Like Synoviocytes From Patients With Rheumatoid Arthritis. Arthritis Rheum (1999) 42:1508–16. doi: 10.1002/1529-0131(199907)42:7<1508::AID-ANR26>3.0.CO;2-L 10403280

[9] Timmer TCG Baltus B Vondenhoff M Huizinga TWJ Tak PP Verweij CL. Inflammation and Ectopic Lymphoid Structures in Rheumatoid Arthritis Synovial Tissues Dissected by Genomics Technology: Identification of the Interleukin-7 Signaling Pathway in Tissues With Lymphoid Neogenesis. Arthritis Rheum (2007) 56:2492–502. doi: 10.1002/art.22748 17665400

[10] Rosengren S Corr M Firestein GS Boyle DL. The JAK Inhibitor CP-690,550 (Tofacitinib) Inhibits TNF-Induced Chemokine Expression in Fibroblast-Like Synoviocytes: Autocrine Role of Type I Interferon. Ann Rheum Dis (2012) 71:440–7. doi: 10.1136/ard.2011.150284 22121136

[11] Bustamante MF Garcia-Carbonell R Whisenant KD Guma M. Fibroblast-Like Synoviocyte Metabolism in the Pathogenesis of Rheumatoid Arthritis. Arthritis Res Ther (2017) 19:1–12. doi: 10.1186/s13075-017-1303-3 28569176 PMC5452638

[12] Tang Y Wang B Sun X Li H Ouyang X Wei J. Rheumatoid Arthritis Fibroblast-Like Synoviocytes Co-Cultured With PBMC Increased Peripheral CD4+CXCR5+ICOS+ T Cell Numbers. Clin Exp Immunol (2017) 190:384–93. doi: 10.1111/cei.13025 PMC5680054 28833034

[13] Liu R Zhao P Zhang Q Che N Xu L Qian J. Adiponectin Promotes Fibroblast-Like Synoviocytes Producing IL-6 to Enhance T Follicular Helper Cells Response in Rheumatoid Arthritis. Clin Exp Rheumatol (2020) 38:11–8. 31025923

[14] Tang BX You X Zhao LD Li Y Zhang X Tang FL. P53 in Fibroblast-Like Synoviocytes can Regulate T Helper Cell Functions in Patients With Active Rheumatoid Arthritis. Chin Med J (Engl) (2011) 124:364–8. doi: 10.3760/cma.j.issn.0366-6999.2011.03.008 21362334

[15] Gao S Qi X Li J Sang L. Upregulated KAT7 in Synovial Fibroblasts Promotes Th17 Cell Differentiation and Infiltration in Rheumatoid Arthritis. Biochem Biophys Res Commun (2017) 489:235–41. doi: 10.1016/j.bbrc.2017.05.143 28552525

[16] Lee DG Woo JW Kwok SK La CM Park SH. MRP8 Promotes Th17 Differentiation *via* Upregulation of IL-6 Production by Fibroblast-Like Synoviocytes in Rheumatoid Arthritis. Exp Mol Med (2013) 45:1–9. doi: 10.1038/emm.2013.39 PMC3641402 23619188

[17] Wang B Ma Z Wang M Sun X Tang Y Li M. IL-34 Upregulated Th17 Production Through Increased IL-6 Expression by Rheumatoid Fibroblast-Like Synoviocytes. Mediators Inflammation (2017) 2017:1567120. doi: 10.1155/2017/1567120 PMC5474253 28659662

[18] Lin J Zhou Z Huo R Xiao L Ouyang G Wang L. Cyr61 Induces IL-6 Production by Fibroblast-Like Synoviocytes Promoting Th17 Differentiation in Rheumatoid Arthritis. J Immunol (2012) 188:5776–84. doi: 10.4049/jimmunol.1103201 22547695

[19] Eljaafari A Tartelin M-L Aissaoui H Chevrel G Osta B Lavocat F. Bone Marrow- and Synovium-Derived Mesenchymal Cells Promote Th-17 Cells Through Caspase-1 Activation: Contribution to Rheumatoid Arthritis Chronicity. Arthritis Rheum (2012) 64(7):2147–57. doi: 10.1002/art 22275154

[20] Corvaisier M Delneste Y Jeanvoine H Preisser L Blanchard S Garo E. IL-26 Is Overexpressed in Rheumatoid Arthritis and Induces Proinflammatory Cytokine Production and Th17 Cell Generation. PLoS Biol (2012) 10(9):e1001395.21. doi: 10.1371/journal.pbio.1001395 23055831 PMC3463509

[21] Xiao X Li Y Jiang X Ji X Lu X Yang B. EZH2 Deficiency Attenuates Treg Diff Erentiation in Rheumatoid Arthritis. J Autoimmun (2019) 108:102404. doi: 10.1016/j.jaut.2020.102404 31952907

[22] Wu X Liu Y Jin S Wang M Jiao Y Yang B. Single-Cell Sequencing of Immune Cells From Anticitrullinated Peptide Antibody Positive and Negative Rheumatoid Arthritis. Nat Commun (2021) 12(1):4977. doi: 10.1038/s41467-021-25246-7 34404786 PMC8371160

[23] Hwang SY Kim HY. Expression of IL-17 Homologs and Their Receptors in the Synovial Cells of Rheumatoid Arthritis Patients. Mol Cells (2005) 19:180–4. 15879699

[24] Hirota K Hashimoto M Ito Y Matsuura M Ito H Tanaka M. Autoimmune Th17 Cells Induced Synovial Stromal and Innate Lymphoid Cell Secretion of the Cytokine GM-CSF to Initiate and Augment Autoimmune Arthritis. Immunity (2018) 48:1220–32.e5. doi: 10.1016/j.immuni.2018.04.009 29802020 PMC6024031

[25] Kim EK Kwon JE Lee SY Lee EJ Kim DS Moon SJ. IL-17-Mediated Mitochondrial Dysfunction Impairs Apoptosis in Rheumatoid Arthritis Synovial Fibroblasts Through Activation of Autophagy. Cell Death Dis (2017) 8(1):e2565. doi: 10.1038/cddis.2016.490 PMC5386390 28102843

[26] Shui XL Lin W Mao CW Feng YZ Kong JZ Chen SM. Blockade of IL-17 Alleviated Inflammation in Rat Arthritis and MMP-13 Expression. Eur Rev Med Pharmacol Sci (2017) 21:2329–37. 28617559

[27] Kim KW Kim HR Kim BM La CM Lee SH. Th17 Cytokines Regulate Osteoclastogenesis in Rheumatoid Arthritis. Am J Pathol (2015) 185:3011–24. doi: 10.1016/j.ajpath.2015.07.017 26362732

[28] Friday SC Fox DA. Phospholipase D Enzymes Facilitate IL-17- and Tnfα-Induced Expression of Proinflammatory Genes in Rheumatoid Arthritis Synovial Fibroblasts (RASF). Immunol Lett (2016) 174:9–18. doi: 10.1016/j.imlet.2016.04.001 27058440

[29] Fan M Li Y Yao C Liu X Liu X Liu J. Dihydroartemisinin Derivative DC32 Attenuates Collagen-Induced Arthritis in Mice by Restoring the Treg/Th17 Balance and Inhibiting Synovitis Through Down-Regulation of IL-6. Int Immunopharmacol (2018) 65:233–43. doi: 10.1016/j.intimp.2018.10.015 30336338

[30] Liu Z Song L Wang Y Xu P Guo X Yang J. A Novel Fusion Protein Attenuates Collagen–Induced Arthritis by Targeting Interleukin 17A and Tumor Necrosis Factor α. Int J Pharm (2018) 547:72–82. doi: 10.1016/j.ijpharm.2018.05.058 29803792

[31] Kim KW La CM HR K JH Ju MK P HJ Oh. Up-Regulation of Stromal Cell-Derived Factor 1 (CXCL12) Production in Rheumatoid Synovial Fibroblasts Through Interactions With T Lymphocytes: Role of Interleukin-17 and CD40L-CD40 Interaction. Arthritis Rheum (2007) 56:1076–86. doi: 10.1002/art.22439 17393416

[32] Aldridge J Ekwall AKH Mark L Bergström B Andersson K Gjertsson I. T Helper Cells in Synovial Fluid of Patients With Rheumatoid Arthritis Primarily Have a Th1 and a CXCR3+Th2 Phenotype. Arthritis Res Ther (2020) 22:1–11. doi: 10.1186/s13075-020-02349-y 33066816 PMC7566124

[33] Kato H Endres J Fox DA. The Roles of IFN-γ Versus IL-17 in Pathogenic Effects of Human Th17 Cells on Synovial Fibroblasts. Mod Rheumatol (2013) 23:1140–50. doi: 10.1007/s10165-012-0811-x PMC3710715 23306426

[34] Corrigall VM Arastu M Khan S Shah C Fife M Tak PP. Functional IL-2 Receptor β (CD122) and γ (CD132) Chains Are Expressed by Fibroblast-Like Synoviocytes: Activation by IL-2 Stimulates Monocyte Chemoattractant Protein-1 Production. J Immunol (2001) 166:4141–7. doi: 10.4049/jimmunol.1201455 11238664

[35] Schurigt U Pfirschke C Irmler IM Hückel M Gajda M Janik T. Interactions of T Helper Cells With Fibroblast-Like Synoviocytes: Up-Regulation of Matrix Metalloproteinases by Macrophage Migration Inhibitory Factor From Both Th1 and Th2 Cells. Arthritis Rheum (2008) 58:3030–40. doi: 10.1002/art.23904 18821693

[36] Saadatmand S Vos JR Hooning MJ Oosterwijk JC Koppert LB De BGH. Interleukin-21 Induces Migration and Invasion of Fibroblast-Like Synoviocytes From Patients With Rheumatoid Arthritis. Clin Exp Immunol (2016) 184:2–31. doi: 10.1111/cei.12751 PMC4837236 26646950

[37] Lebre MC Vieira PL Tang MW Aarrass S Helder B Newsom-Davis T. Synovial IL-21/TNF-Producing CD4+ T Cells Induce Joint Destruction in Rheumatoid Arthritis by Inducing Matrix Metalloproteinase Production by Fibroblast-Like Synoviocytes. J Leukoc Biol (2017) 101:775–83. doi: 10.1189/jlb.5a0516-217rr 27733582

[38] Blaschke S Middel P Dorner BG Blaschke V Hummel KM Kroczek RA. Expression of Activation-Induced, T Cell-Derived, and Chemokine-Related Cytokine/Lymphotactin and Its Functional Role in Rheumatoid Arthritis. Arthritis Rheum (2003) 48:1858–72. doi: 10.1002/art.11171 12847680

[39] Zhu W Meng L Jiang C He X Hou W Xu P. Arthritis is Associated With T-Cell-Induced Upregulation of Toll-Like Receptor 3 on Synovial Fibroblasts. Arthritis Res Ther (2011) 13(3):R103. doi: 10.1186/ar3384 21708001 PMC3218918

[40] Karonitsch T Beckmann D Dalwigk K Niederreiter B Studenic P Byrne RA. Targeted Inhibition of Janus Kinases Abates Interfon Gamma-Induced Invasive Behaviour of Fibroblast-Like Synoviocytes. Rheumatol (United Kingdom) (2018) 57:572–7. doi: 10.1093/rheumatology/kex426 29228301

[41] Kvacskay P Yao N Schnotz JH Scarpone R Carvalho R de A Klika KD. Increase of Aerobic Glycolysis Mediated by Activated T Helper Cells Drives Synovial Fibroblasts Towards an Inflammatory Phenotype: New Targets for Therapy? Arthritis Res Ther (2021) 23:1–15. doi: 10.1186/s13075-021-02437-7 33588937 PMC7883459

[42] Tran CN Davis MJ Tesmer LA Endres JL Motyl CD Smuda C. Presentation of Arthritogenic Peptide to Antigen-Specific T Cells by Fibroblast-Like Synoviocytes. Arthritis Rheum (2007) 56:1497–506. doi: 10.1002/art.22573 17469112

[43] Riemann D Röntsch J Hause B Langner J Kehlen A. Cell-Cell Contact Between Lymphocytes and Fibroblast-Like Synoviocytes Induces Lymphocytic Expression of Aminopeptidase N/CD13 and Results in Lymphocytic Activation. Adv Exp Med Biol (2000) 477:57–66. doi: 10.1007/0-306-46826-3_6 10849731

[44] Bradfield PF Amft N Vernon-Wilson E Exley AE Parsonage G Rainger GE. Rheumatoid Fibroblast-Like Synoviocytes Overexpress the Chemokine Stromal Cell-Derived Factor 1 (CXCL12), Which Supports Distinct Patterns and Rates of CD4+ and CD8+ T Cell Migration Within Synovial Tissue. Arthritis Rheum (2003) 48:2472–82. doi: 10.1002/art.11219 13130466

[45] Sawai H Park YW Roberson J Imai T Goronzy JJ Weyand CM. T Cell Costimulation by Fractalkine-Expressing Synoviocytes in Rheumatoid Arthritis. Arthritis Rheum (2005) 52:1392–401. doi: 10.1002/art.21140 15880821

[46] Vallejo AN Yang H Klimiuk PA Weyand CM Goronzy JJ. Synoviocyte-Mediated Expansion of Inflammatory T Cells in Rheumatoid Synovitis Is Dependent on CD47-Thrombospondin 1 Interaction. J Immunol (2003) 171:1732–40. doi: 10.4049/jimmunol.171.4.1732 12902472

[47] Vallejo AN Mügge LO Klimiuk PA Weyand CM Goronzy JJ. Central Role of Thrombospondin-1 in the Activation and Clonal Expansion of Inflammatory T Cells. J Immunol (2000) 164:2947–54. doi: 10.4049/jimmunol.164.6.2947 10706681

[48] Corrigall VM Solau-Gervais E Panayi GS. Lack of CD80 Expression by Fibroblast-Like Synoviocytes Leading to Anergy in T Lymphocytes. Arthritis Rheum (2000) 43:1606–15. doi: 10.1002/1529-0131(200007)43:7<1606::AID-ANR26>3.0.CO;2-O 10902766

[49] Tran CN Thacker SG Louie DM Oliver J White PT Endres JL. Interactions of T Cells With Fibroblast-Like Synoviocytes: Role of the B7 Family Costimulatory Ligand B7-H3. J Immunol (2008) 180:2989–98. doi: 10.4049/jimmunol.180.5.2989 18292521

[50] Kim SJ Shin HH Park SY Lee DS Lee EA Cho SD. Induction of MMP-13 Expression by Soluble Human Glucocorticoid-Induced Tumor Necrosis Factor Receptor in Fibroblast-Like Synovial Cells. Osteoarthr Cartil (2006) 14:146–53. doi: 10.1016/j.joca.2005.08.012 16242974

[51] Min DJ Cho ML SH L SY M WU K JK M. Augmented Production of Chemokines by the Interaction of Type II Collagen-Reactive T Cells With Rheumatoid Synovial Fibroblasts. Arthritis Rheum (2004) 50:1146–55. doi: 10.1002/art.20133 15077296

[52] Sawai H Park YW He X Goronzy JJ Weyand CM. Fractalkine Mediates T Cell-Dependent Proliferation of Synovial Fibroblasts in Rheumatoid Arthritis. Arthritis Rheum (2007) 56:3215–25. doi: 10.1002/art.22919 17907166

[53] Petrasca A Phelan JJ Ansboro S Veale DJ Fearon U Fletcher JM. Targeting Bioenergetics Prevents CD4 T Cell-Mediated Activation of Synovial Fibroblasts in Rheumatoid Arthritis. Rheumatol (United Kingdom) (2020) 59:2816–28. doi: 10.1093/rheumatology/kez682 32047926

[54] Singh K Colmegna I He X Weyand CM Goronzy JJ. Synoviocyte Stimulation by the LFA-1–Intercellular Adhesion Molecule-2–Ezrin–Akt Pathway in Rheumatoid Arthritis. J Immunol (2008) 180:1971–8. doi: 10.4049/jimmunol.180.3.1971 18209096

[55] Tran CN Lundy SK White PT Endres JL Motyl CD Gupta R. Molecular Interactions Between T Cells and Fibroblast-Like Synoviocytes: Role of Membrane Tumor Necrosis Factor-α on Cytokine-Activated T Cells. Am J Pathol (2007) 171:1588–98. doi: 10.2353/ajpath.2007.070004 PMC2043519 17823284

[56] Bombara MP Webb DL Conrad P Marlor CW Sarr T Ranges GE. Cell Contact Between T Cells and Synovial Fibroblasts Causes Induction of Adhesion Molecules and Cytokines. J Leukoc Biol (1993) 54:399–406. doi: 10.1002/jlb.54.5.399 7693840

[57] Cho ML Yoon CH Hwang SY Park MK Min SY Lee SH. Effector Function of Type II Collagen-Stimulated T Cells From Rheumatoid Arthritis Patients: Cross-Talk Between T Cells and Synovial Fibroblasts. Arthritis Rheum (2004) 50:776–84. doi: 10.1002/art.20106 15022319

[58] Moon YM Yoon BY Her YM Oh HJ Lee JS Kim KW. IL-32 and IL-17 Interact and Have the Potential to Aggravate Osteoclastogenesis in Rheumatoid Arthritis. Arthritis Res Ther (2012) 14(6):R246. doi: 10.1186/ar4089 23148681 PMC3674587

[59] Tanner MR Pennington MW Chauhan SS Laragione T Gulko PS Beeton C. KCa1.1 and Kv1.3 Channels Regulate the Interactions Between Fibroblast-Like Synoviocytes and T Lymphocytes During Rheumatoid Arthritis. Arthritis Res Ther (2019) 21:1–21. doi: 10.1186/s13075-018-1783-9 30612588 PMC6322314

[60] Akaogi J Nozaki T Satoh M Yamada H. Role of PGE2 and EP Receptors in the Pathogenesis of Rheumatoid Arthritis and as a Novel Therapeutic Strategy. Endocrine Metab Immune Disord - Drug Targets (2012) 6:383–94. doi: 10.2174/187153006779025711 17214584

[61] Zhang W Wang B Wang F Zhang J Yu J. CTLA4-FasL Fusion Product Suppresses Proliferation of Fibroblast-Like Synoviocytes and Progression of Adjuvant-Induced Arthritis in Rats. Mol Immunol (2012) 50:150–9. doi: 10.1016/j.molimm.2012.01.007 22325471

[62] Kraan MC Haringman JJ Weedon H Barg EC Smith MD Ahern MJ. T Cells, Fibroblast-Like Synoviocytes, and Granzyme B+ Cytotoxic Cells are Associated With Joint Damage in Patients With Recent Onset Rheumatoid Arthritis. Ann Rheum Dis (2004) 63:483–8. doi: 10.1136/ard.2003.009225 PMC1755001 15082476

[63] Brooke A Bs R Mph CH Ma MC Shao X Ba HLQ. Rheumatoid Arthritis Naïve T Cells Share Hypermethylation Sites With Synoviocytes. Arthritis Rheumatol (2017) 69:550–9. doi: 10.1002/art PMC5328845 27723282

[64] Dinesh P Rasool MK. Berberine Mitigates IL-21/IL-21R Mediated Autophagic Influx in Fibroblast-Like Synoviocytes and Regulates Th17/Treg Imbalance in Rheumatoid Arthritis. Apoptosis (2019) 24:644–61. doi: 10.1007/s10495-019-01548-6 31111379

[65] Tong WW Zhang C Hong T Liu DH Wang C Li J. Silibinin Alleviates Inflammation and Induces Apoptosis in Human Rheumatoid Arthritis Fibroblast-Like Synoviocytes and has a Therapeutic Effect on Arthritis in Rats. Sci Rep (2018) 8:1–12. doi: 10.1038/s41598-018-21674-6 29459717 PMC5818498

[66] Park YH Jang YJ Choi Y Lee K Kim HJ Cho O. Combination of LMT-28 and Metformin Improves Beneficial Anti-Inflammatory Effect in Collagen-Induced Arthritis. Pharmacology (2021) 106:53–9. doi: 10.1159/000507451 32674107

[67] Park YH Kim HJ Heo TH. A Directly GP130-Targeting Small Molecule Ameliorates Collagen-Induced Arthritis (CIA) by Inhibiting IL-6/GP130 Signalling and Th17 Differentiation. Clin Exp Pharmacol Physiol (2020) 47:628–39. doi: 10.1111/1440-1681.13215 31742738

[68] Park Y Jung H Lee K Choi Y Heo T. Combination of Gp130-Targeting and TNF-Targeting Small Molecules in Alleviating Arthritis Through the Down-Regulation of Th17 Differentiation and Osteoclastogenesis. Biochem Biophys Res Commun (2019) 522(4):1030–36. doi: 10.1016/j.bbrc.2019.11.183 31818460

[69] Liang JJ Li HR Chen Y Zhang C Chen DG Liang ZC. Diallyl Trisulfide can Induce Fibroblast-Like Synovial Apoptosis and has a Therapeutic Effect on Collagen-Induced Arthritis in Mice *via* Blocking NF-κb and Wnt Pathways. Int Immunopharmacol (2019) 71:132–8. doi: 10.1016/j.intimp.2019.03.024 30897500

[70] Wang YL Gao JM Xing LZ. Therapeutic Potential of Oroxylin A in Rheumatoid Arthritis. Int Immunopharmacol (2016) 40:294–9. doi: 10.1016/j.intimp.2016.09.006 27643663

[71] Odobasic D Jia Y Kao W Fan H Wei X Gu R. Formyl Peptide Receptor Activation Inhibits the Expansion of Effector T Cells and Synovial Fibroblasts and Attenuates Joint Injury in Models of Rheumatoid Arthritis. Int Immunopharmacol (2018) 61:140–9. doi: 10.1016/j.intimp.2018.05.028 29879657

[72] Spurlock CF Gass HM Bryant CJ Wells BC Olsen NJ Aune TM. Methotrexate-Mediated Inhibition of Nuclear Factor κb Activation by Distinct Pathways in T Cells and Fibroblast-Like Synoviocytes. Rheumatol (United Kingdom) (2014) 54:178–87. doi: 10.1093/rheumatology/keu279 PMC4269792 25118313

[73] Wang Y Wu Q Liu Z Guo X Zhou L Wang Y. A Recombinant IgG-Like Bispecific Antibody Acting as Interleukin-1β and Interleukin-17A Inhibitor Exhibits a Promising Efficacy for Rheumatoid Arthritis. BioMed Pharmacother (2017) 89:426–37. doi: 10.1016/j.biopha.2017.02.045 28249243

[74] Liu Y Mu R Wang S Long L Liu X Li R. Therapeutic Potential of Human Umbilical Cord Mesenchymal Stem Cells in the Treatment of Rheumatoid Arthritis. Arthritis Res Ther (2010) 12:1–13. doi: 10.1186/ar3187 PMC3046518 21080925

[75] Papadopoulou A Yiangou M Athanasiou E Zogas N Kaloyannidis P Batsis I. Mesenchymal Stem Cells are Conditionally Therapeutic in Preclinical Models of Rheumatoid Arthritis. Ann Rheum Dis (2012) 71:1733–40. doi: 10.1136/annrheumdis-2011-200985 22586171

[76] Kang SE Park JK Yoo HJ Kang HS Park YW Park BC. Efficacy of Novel Bispecific Antibody Targeting TNF-α/CXCL10 in the Treatment of Experimental Arthritis. Transl Res (2021) 232:75–87. doi: 10.1016/j.trsl.2021.01.004 33453429

[77] Chen H Wang C Li J Huandike M Liu J Huang Q. Chinese Herbal Formula, Huayu Tongbi Fang, Attenuates Inflammatory Proliferation of Rat Synoviocytes Induced by IL-1 β by Regulating Proliferation and Differentiation of T Lymphocytes. Evidence-Based Complement Altern Med (2020) 2020:1706837. doi: 10.1155/2020/1706837 PMC7256709 32565847

[78] Matsuoka N Eguchi K Kawakami A Tsuboi M Kawabe Y Aoyagi T. Inhibitory Effect of Clarithromycin on Costimulatory Molecule Expression and Cytokine Production by Synovial Fibroblast-Like Cells. Clin Exp Immunol (1996) 104:501–8. doi: 10.1046/j.1365-2249.1996.46752.x PMC2200457 9099936

[79] Zhu Q Huang J Wang SZ Qin ZH Lin F. Cobrotoxin Extracted From Naja Atra Venom Relieves Arthritis Symptoms Through Anti-Inflammation and Immunosuppression Effects in Rat Arthritis Model. J Ethnopharmacol (2016) 194:1087–95. doi: 10.1016/j.jep.2016.11.009 27840083

[80] Kwok SK Cho ML Park MK Oh HJ Park JS Her YM. Interleukin-21 Promotes Osteoclastogenesis in Humans With Rheumatoid Arthritis and in Mice With Collagen-Induced Arthritis. Arthritis Rheum (2012) 64:740–51. doi: 10.1002/art.33390 21968544

[81] Kotake S Udagawa N Hakoda M Mogi M Yano K Tsuda E. Activated Human T Cells Directly Induce Osteoclastogenesis From Human Monocytes: Possible Role of T Cells in Bone Destruction in Rheumatoid Arthritis Patients. Arthritis Rheum (2001) 44:1003–12. doi: 10.1002/1529-0131(200105)44:5<1003::aid-anr179>3.0.co;2-# 11352231

[82] Guo W Yu D Wang X Luo C Chen Y Lei W. Anti-Inflammatory Effects of Interleukin-23 Receptor Cytokinebinding Homology Region Rebalance T Cell Distribution in Rodent Collagen-Induced Arthritis. Oncotarget (2016) 7:31800–13. doi: 10.18632/oncotarget.9309 PMC5077977 27177334

[83] Shang ZZ Qin DY Li QM Zha XQ Pan LH Peng DY. Dendrobium Huoshanense Stem Polysaccharide Ameliorates Rheumatoid Arthritis in Mice *via* Inhibition of Inflammatory Signaling Pathways. Carbohydr Polym (2021) 58(3):718–29. doi: 10.1016/j.carbpol.2021.117657 33593544

[84] Seong AR Yoo JY Choi KC Lee MH Lee YH Lee J. Delphinidin, a Specific Inhibitor of Histone Acetyltransferase, Suppresses Inflammatory Signaling *via* Prevention of NF-κb Acetylation in Fibroblast-Like Synoviocyte MH7A Cells. Biochem Biophys Res Commun (2011) 410:581–6. doi: 10.1016/j.bbrc.2011.06.029 21683061

[85] Jia X Wei F Sun X Chang Y Xu S Yang X. CP-25 Attenuates the in Flammatory Response of Fibroblast-Like Synoviocytes Co-Cultured With BAFF-Activated CD4+ T Cells. J Ethnopharmacol J (2016) 189:194–201. doi: 10.1016/j.jep.2016.05.034 27196292

[86] Zhang G Liu HB Zhou L Cui XQ Fan XH. CCL3 Participates in the Development of Rheumatoid Arthritis by Activating AKT. Eur Rev Med Pharmacol Sci (2018) 22:6625–32. doi: 10.26355/eurrev_201810_16137 30402834

[87] Vandooren B Cantaert T Noordenbos T Tak PP Baeten D. The Abundant Synovial Expression of the RANK/RANKL/osteoprotegerin System in Peripheral Spondylarthritis is Partially Disconnected From Inflammation. Arthritis Rheum (2008) 58:718–29. doi: 10.1002/art.23290 18311801

[88] Fitch E Harper E Skorcheva I Kurtz SE Blauvelt A. Pathophysiology of Psoriasis: Recent Advances on IL-23 and Th17 Cytokines. Curr Rheumatol R (2007) 9:461–7. doi: 10.1007/s11926-007-0075-1 PMC2893221 18177599

[89] Mathieu MC Lord-Dufour S Bernier V Boie Y Burch JD Clark P. Mutual Antagonistic Relationship Between Prostaglandin E2 and IFN-γ: Implications for Rheumatoid Arthritis. Eur J Immunol (2008) 38:1900–12. doi: 10.1002/eji.200838170 18506884

